# Lister’s Antiseptic Method in Ovariotomy

**Published:** 1877-01

**Authors:** 


Lister's Antiseptic Method in Ovariotomy.—Dr. J. Marion Sims, of New York, contributes to the *Medical Record*, a case of the application of the antiseptic method to this operation; the first, it is believed, on record.

The patient, forty-seven years of age, noticed a tumor the size of an orange, in the right iliac region, last April. This was pronounced to be an ovarian tumor. Dr. Thomas, of New York, and Dr. Atlee, of Philadelphia, were consulted, and agreed in the diagnosis previously made by her family physician, but both declined to operate while the tumor occasioned so little trouble. Dr. Sims was consulted September 20th, and also declined to operate, notwithstanding the entreaties of the patient. Vomiting, emaciation and debility, with a slight attack of peritonitis, made their appearance early in November, hence Dr. Sims concluded to defer the operation no longer. This was done on November 23d last. Dr. Sass directed the spray, from his new apparatus, which covered the seat of the operation with a delicate carbolic mist. The hands, sponges and instruments were all dipped in carbolic water. The operation and dressing lasted forty minutes, the spray being kept up all the time. It (the spray) could have been continued two hours, if necessary. There were no adhesions. The peritoneal cavity contained six or eight ounces of a reddish serum. The peritoneal membrane was everywhere deeply congested. This fact explains the presence of reddish serum, and the previous attack of peritonitis.

The pedicle was very short, and at least three inches broad. It was tied in three sections with strong twine, and drawn out and fixed in the lower angle of the wound, clamp-fashion.

The external incision was closed by sutures, and a carbolized dressing applied.

The pulse never rose above 90, nor the temperature over 101°.

Convalescence was fully assured in forty-eight hours, and the patient is now quite well. The tumor was polycistic, on right side, and weighed fifteen pounds.

Dr. Sims thinks that Lister's antiseptic method will prove as valuable in ovariotomy as it has in general surgery.

He bears testimony to the great value of Dr. Sass' spray apparatus, and thinks he has rendered the profession a great service by bringing it forward at this time.

